# Intelligent and precise auxiliary diagnosis of breast tumors using deep learning and radiomics

**DOI:** 10.1371/journal.pone.0320732

**Published:** 2025-06-02

**Authors:** Ting Wang, Boyang Zang, Chui Kong, Yigang Li, Xiaomin Yang, Yi Yu

**Affiliations:** 1 XinHua Hospital Affiliated to Shanghai Jiao Tong University School of Medicine Cardiology, Shanghai, 200092, China; 2 School of Clinical Medicine, Tsinghua University, Beijing, 100084, China; 3 School of Information Science and Technology, Fudan University, Shanghai, 200433, China; 4 Department of Ultrasound, Shanghai Chest Hospital, Shanghai Jiao Tong University School of Medicine, 200030, China; Manchester Metropolitan University, UNITED KINGDOM OF GREAT BRITAIN AND NORTHERN IRELAND

## Abstract

**Background:**

Breast cancer is the most common malignant tumor among women worldwide, and early diagnosis is crucial for reducing mortality rates. Traditional diagnostic methods have significant limitations in terms of accuracy and consistency. Imaging is a common technique for diagnosing and predicting breast cancer, but human error remains a concern. Increasingly, artificial intelligence (AI) is being employed to assist physicians in reducing diagnostic errors.

**Methods:**

We developed an intelligent diagnostic model combining deep learning and radiomics to enhance breast tumor diagnosis. The model integrates MobileNet with ResNeXt-inspired depthwise separable and grouped convolutions, improving feature processing and efficiency while reducing parameters. Using AI-Dhabyani and TCIA breast ultrasound datasets, we validated the model internally and externally, comparing it to VGG16, ResNet, AlexNet, and MobileNet. Results: The internal validation set achieved an accuracy of 83.84% with an AUC of 0.92, outperforming other models. The external validation set showed an accuracy of 69.44% with an AUC of 0.75, demonstrating high robustness and generalizability. Conclusions: We developed an intelligent diagnostic model using deep learning and radiomics to improve breast tumor diagnosis. The model combines MobileNet with ResNeXt-inspired depthwise separable and grouped convolutions, enhancing feature processing and efficiency while reducing parameters. It was validated internally and externally using the AI-Dhabyani and TCIA breast ultrasound datasets and compared with VGG16, ResNet, AlexNet, and MobileNet.

## 1 Background

Breast cancer is one of the most common malignant tumors among women globally and a leading cause of cancer-related deaths in women. According to recent statistics, approximately 2 million women are diagnosed with breast cancer annually, with about 600,000 deaths attributed to the disease [1,2]. Early diagnosis and treatment are crucial for reducing the mortality rate of breast cancer. However, traditional diagnostic methods such as mammography, clinical breast examination, and biopsy are subjective, have high misdiagnosis rates, and their accuracy is influenced by the radiologist’s experience [3]. These issues underscore the need for more accurate and reliable diagnostic tools to support radiologists in making precise clinical decisions.

Recent advancements in deep learning and medical imaging technologies offer new hope for addressing these challenges [4]. Convolutional neural networks (CNNs), in particular, have demonstrated outstanding performance in image classification, detection, and segmentation tasks [5]. Some studies have applied deep learning techniques to breast ultrasound imaging to improve the detection and classification accuracy of breast tumors [6,7]. Breast ultrasound, as a non-invasive and cost-effective imaging modality, holds significant advantages in breast cancer screening, especially in women with dense breast tissue where ultrasound sensitivity surpasses that of mammography [8]. However, the quality and interpretability of ultrasound images vary greatly, posing additional challenges for automated analysis [9].

The integration of radiomics and deep learning has shown tremendous potential in enhancing the accuracy of medical imaging diagnostics [10]. Radiomics involves extracting quantitative features from medical images, capturing tumor heterogeneity, and providing additional diagnostic and prognostic information [11]. When combined with deep learning models, these features can significantly improve the performance of classification algorithms [12]. Recent studies exploring the application of radiomics in breast cancer diagnosis have demonstrated high accuracy in distinguishing between benign and malignant lesions [13,14]. However, further comprehensive research is needed to validate these methods across diverse patient populations and imaging conditions.

**Fig 1:**
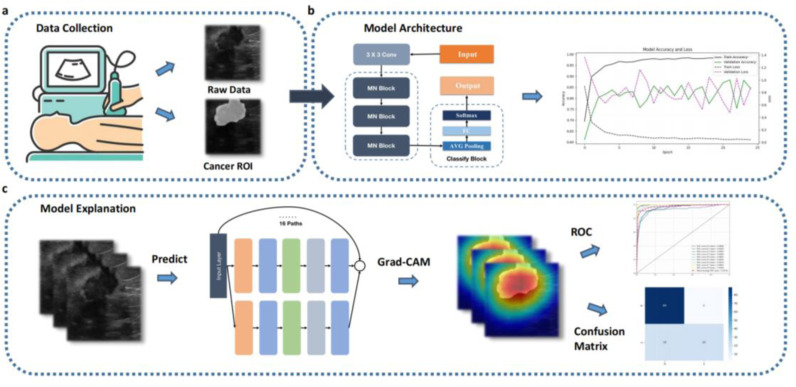
Workflow: (a) data collection, (b) network training, and (c) network validation.

This study aims to develop an intelligent and precise auxiliary diagnostic model for breast tumors based on deep learning and radiomics, with external validation using independent datasets (Fig 1). We utilized two publicly available breast ultrasound image datasets, containing images from patients aged 25–75, annotated by experienced radiologists [15,16]. Our model combines the MobileNet architecture with Next Convolution Block (NCB) technology and is compared with advanced models such as VGG16, ResNet, DenseNet, MobileNet, and AlexNet. Performance metrics including accuracy, precision, recall, F1 score, and AUC are used to evaluate these models, with validation on an external test set. The results demonstrate that our proposed model exhibits superior performance in both internal and external validations, highlighting its potential for clinical application. This study aims to improve the accuracy and efficiency of breast cancer diagnosis, supporting radiologists in making more accurate and efficient clinical decisions, ultimately improving patient outcomes (Fig 1).

The rest of this paper is organized as follows. Section 2 describes the datasets and preprocessing procedures employed in this study. Section 3 introduces the architecture of the proposed model and its baseline counterparts, followed by an evaluation of the model’s performance based on various metrics. Section 4 presents the results of the experiments, including internal and external validation, data augmentation, and the incorporation of lesion segmentation information. Finally, Section 5 discusses the clinical interpretability of the proposed model, its practical implications, and potential future research directions.

## 2 Methods

### 2.1 Database

The data used in this study were obtained from two publicly available breast ultrasound image datasets. The first dataset was published by AI-Dhabyani et al. in 2020, containing 780 breast ultrasound images from 600 female patients aged 25–75 years [15]. The images, with a resolution of 500x500 pixels, are categorized into normal, benign, and malignant classes. This dataset is available at https://www.ncbi.nlm.nih.gov/pmc/articles/PMC6906728/. The second dataset is from The Cancer Imaging Archive (TCIA), comprising 256 ultrasound scans from 256 patients, including 266 segments of benign and malignant lesions [16]. Each image in this dataset has been manually annotated by experienced radiologists according to the Breast Imaging-Reporting and Data System (BI-RADS) standards, providing detailed patient-level and tumor-level labels. This dataset is accessible at https://www.cancerimagingarchive.net/collection/breast-lesions-usg/. All cases in both datasets were confirmed through follow-up care or biopsy results.

### 2.2 Data Preprocessing

In this study, we applied a unified preprocessing procedure to all breast ultrasound images to ensure data consistency and model effectiveness. All images were resized to 256x256 pixels to standardize the image dimensions. A fixed region of interest (ROI) of 224x224 pixels was then selected from each image. This approach helps to focus on the breast lesion area, reducing background noise and enhancing the efficiency and accuracy of model training.

The preprocessed data were divided into training and testing sets for model training and validation. The training set was used to train the model, while the testing set was employed for internal validation to evaluate the model’s performance on unseen data.

To enhance the model’s robustness, we applied data augmentation techniques to the training set, increasing the diversity of the training data. These techniques included image rotation (randomly within a range of ±15°), horizontal and vertical flipping, and jittering (adjusting brightness, contrast, saturation, and hue within a range of ±10%). By generating a more varied set of training samples with controlled parameters, we aimed to improve the model’s generalization capabilities and reduce overfitting.


$$\begin{array}{c} pixel\;=(1-a)\times pixel1+(1-a)\times pixel1 \end{array}$$ (1)

**Fig 2:**
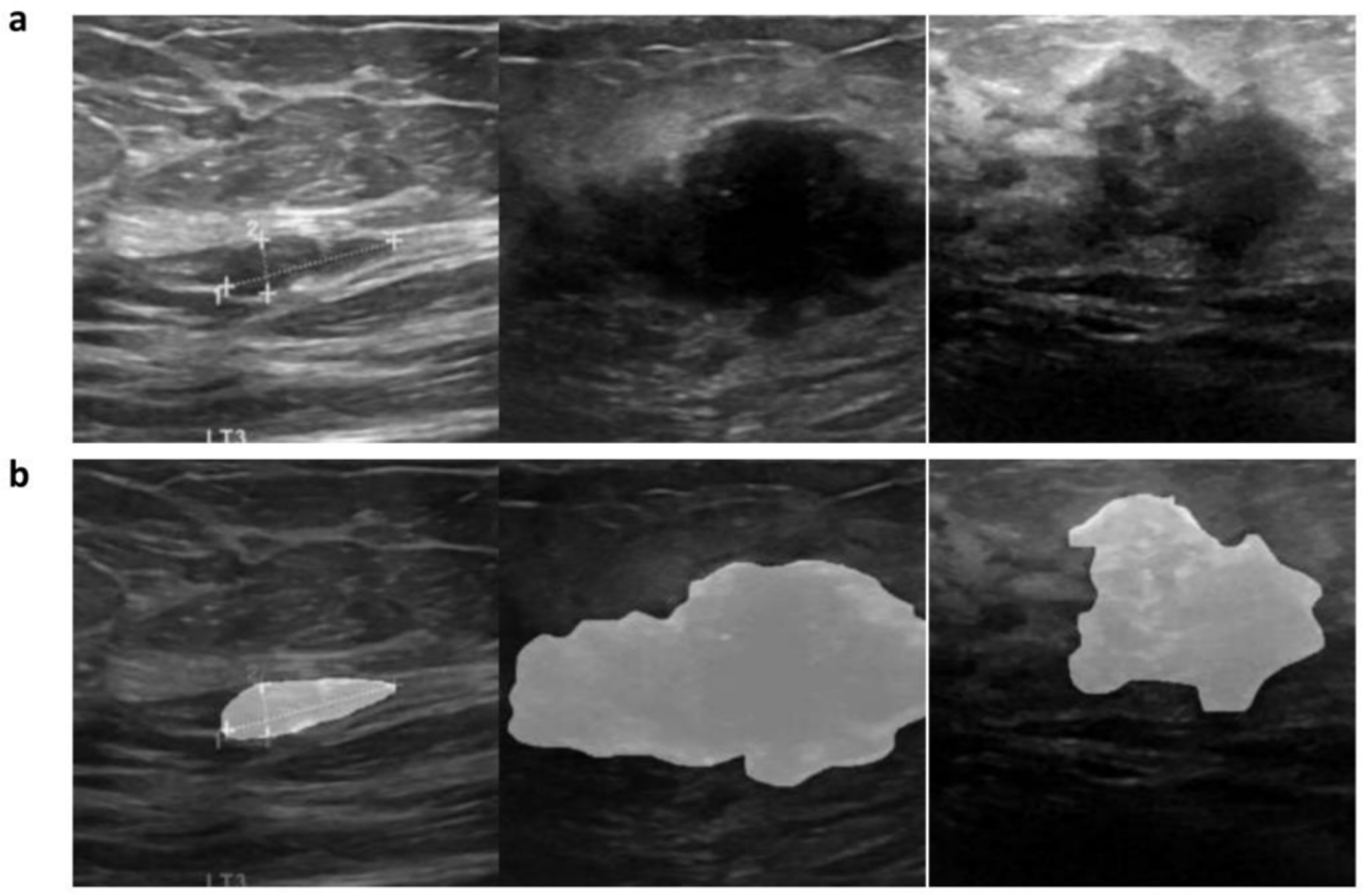
Data Annotation: Radiologist-Segmented Lesion ROIs. a. The first column shows the original ultrasound images. b. The second column displays the images with integrated lesion ROI (Region of Interest) results.

Additionally, to further improve model performance, we incorporated the radiologists’ segmentation information of the lesions into the original images. During the training process, this was achieved by interpolating and blending the segmented lesion areas with the original images, as shown in Equation (1). This preprocessing step enhanced the breast cancer recognition features and provided a reference for future research on breast cancer detection under image segmentation. See Fig 2 for details.

### 2.3 Model Construction

#### 2.3.1 Proposed Models.

In the field of deep learning, convolutional neural networks (CNNs) have been widely applied to medical image analysis, achieving significant results [4]. Classic models such as VGG16, ResNet, MobileNet, AlexNet, and DenseNet have shown excellent performance in image classification tasks, yet each has its limitations.

VGG16 enhances performance by increasing network depth, but this comes with high computational complexity and extended training times [17]. ResNet addresses the vanishing gradient problem in deep networks through residual connections, but its complex structure demands substantial hardware resources [17]. MobileNet reduces the number of parameters and computational load through depthwise separable convolutions, but this simplification can slightly compromise accuracy [18]. AlexNet, an earlier deep learning model, achieved great success on ImageNet but falls short in performance and efficiency compared to more modern models [20]. DenseNet alleviates the vanishing gradient problem through dense connectivity, but this results in increased memory consumption due to the significant number of connections [19].

Given these limitations, our study proposes a novel hybrid model that combines MobileNet and ResNeXt. By integrating depthwise convolutions and grouped convolutions, we aim to construct a new network model that maintains accuracy while optimizing computational efficiency and adaptability. This approach seeks to leverage the strengths of both architectures, addressing the specific challenges posed by breast ultrasound image analysis.

#### 2.3.2 Baseline Models.

Our proposed model combines the lightweight structure of MobileNet with the grouped convolution modules of ResNeXt. MobileNet’s depthwise separable convolutions significantly reduce the number of parameters and computational load. Traditional convolution operations perform spatial and channel convolutions simultaneously, whereas depthwise separable convolutions decompose this process into depthwise convolution and pointwise convolution, making it suitable for resource-limited medical scenarios [18]. ResNeXt, an improved version of ResNet, enhances model performance by increasing network width. It primarily employs a technique called “grouped convolution” to expand network width and introduces the concept of “cardinality” to control this width [17].

**Fig 3:**
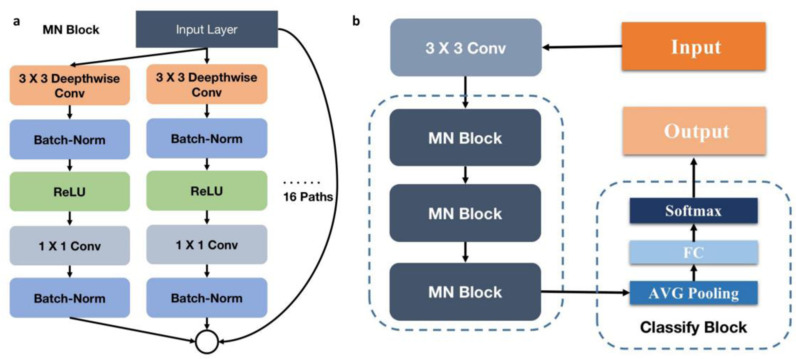
a. MN Block is used in the network. b. Model Architecture Diagram.

In our model architecture, we use MobileNet as the base network and integrate multiple ResNeXt modules to enhance both the depth and width of the model. Specifically, we replace the convolution modules in MobileNet with ResNeXt grouped convolution modules. Each module employs depthwise separable convolutions, leveraging grouped convolutions to increase network width while reducing model parameters and enhancing computational efficiency. Our architecture consists of three such modules, each with 16 paths, followed by average pooling and a fully connected layer, culminating in softmax classification. This structure effectively balances accuracy and efficiency, as illustrated in Fig 3.

### 2.4 Model Evaluation

Evaluation metrics are critical for assessing the performance of machine learning models. These measures are essential for objectively evaluating the models’ performance and guiding their development and improvement. In this experiment, we used several metrics to evaluate the effectiveness of each model. These metrics include accuracy, precision, recall, F1 score, and the area under the receiver operating characteristic curve (AUC). Accuracy measures the percentage of correctly predicted results out of the total samples. Precision indicates the probability that a sample predicted as positive is actually positive. Recall reflects the probability that a sample which is actually positive is predicted as positive. The F1 score is a comprehensive measure that balances precision and recall. The ROC curve, derived from the confusion matrix, is used to evaluate the model’s predictive capability, with AUC representing the area under the ROC curve. This study uses these metrics to provide a robust evaluation of the model’s performance.

## 3 Results

### 3.1 Experimental Setup

In our experiments, we meticulously set up the evaluation of the model’s performance in breast cancer diagnosis. The dataset was divided into training and testing sets. The AI-Dhabyani dataset served as the internal test set, with 80% used for training and 20% for testing. The TCIA dataset was used for external validation to ensure accurate performance assessment.

All tasks were performed on a Windows 10 system equipped with an AMD Ryzen 7 5800H CPU (16 GB RAM) and a GeForce RTX™ 3090 GPU (24 GB RAM). We used Python 3.10 and PyTorch 1.9.0 for model construction and training.

To achieve optimal performance, we fine-tuned all model parameters, employing Stochastic Gradient Descent (SGD) and Adaptive Moment Estimation (Adam) optimization algorithms with a learning rate of 0.00001 and default momentum parameters (beta1 = 0.9, beta2 = 0.999). These optimization algorithms were chosen for their complementary advantages: SGD’s stability in convergence and Adam’s adaptive learning rates for efficient optimization in complex settings. Training was conducted over 50 epochs with a learning rate decay strategy, reducing the learning rate by a factor of 0.1 at the end of each epoch, to facilitate gradual convergence and prevent overfitting. The batch size was set to 32, balancing memory constraints and convergence stability. Additionally, we applied L2 regularization to penalize large weights and Dropout techniques to mitigate overfitting, both enhancing model generalization and stability. These hyperparameters were determined through empirical experiments to ensure the best trade-off between accuracy and computational efficiency.

### 3.2 The Results of Models

#### 3.2.1 Baseline.

**Fig 4:**
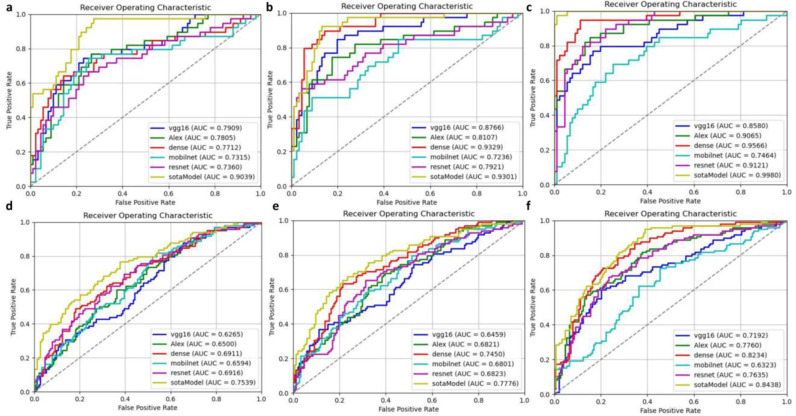
Testing results. The first row a, b, c represents the internal validation results in Baseline, Data Augmentation, and Addition of Lesion ROI, respectively. The second row d, e, f represents the external validation results in Baseline, Data Augmentation, and Addition of Lesion ROI, respectively.

In our experiments, the proposed state-of-the-art (SOTA) model outperformed other models across key metrics. In internal validation, it achieved an AUC of 0.9039, accuracy of 85.38%, and precision of 0.9545, surpassing VGG16, AlexNet, DenseNet, MobileNet, and ResNet. In external validation, the SOTA model also showed strong performance with an AUC of 0.7539 and precision of 0.8571, outperforming VGG16 and AlexNet. While models like DenseNet and ResNet had higher AUC, the SOTA model demonstrated superior precision and overall robustness, proving its effectiveness for breast tumor diagnosis.(see Table 1, Fig 4).

**Table 1:** The model performance of the baseline.

Test	Model	AUC	Accuracy	F1	Precision	Recall
Internal	vgg16	0.7909	0.8000	0.6389	0.6970	0.5897
	Alex	0.7805	0.7692	0.5714	0.6452	0.5128
	dense	0.7712	0.8077	0.6377	0.7333	0.5641
	mobilnet	0.7315	0.7615	0.4561	0.7222	0.3333
	resnet	0.7360	0.7692	0.5455	0.6667	0.4615
	**sotaModel**	**0.9039**	**0.8538**	**0.6885**	**0.9545**	**0.5385**
External	vgg16	0.6265	0.6508	0.3714	0.6190	0.2653
	Alex	0.6500	0.6270	0.3286	0.5476	0.2347
	dense	0.6911	0.6627	0.4654	0.6066	0.3776
	mobilnet	0.6594	0.6468	0.2645	0.6957	0.1633
	resnet	0.6916	0.6706	0.4970	0.6119	0.4184
	**sotaModel**	**0.7539**	**0.6905**	**0.3810**	**0.8571**	**0.2449**

#### 3.2.2 Data Augmentation.

After applying image augmentation, our proposed model showed improvements across all metrics. The proposed state-of-the-art (SOTA) model outperformed other models in both internal and external validation. Internally, it achieved an AUC of 0.9301, accuracy of 86.92%, and precision of 0.7750, surpassing VGG16, AlexNet, MobileNet, and ResNet. While DenseNet had the highest AUC of 0.9329, the SOTA model showed better precision and recall, demonstrating superior overall performance. Externally, it achieved an AUC of 0.7776 and precision of 0.7143, outperforming VGG16 and AlexNet. Though DenseNet had a higher AUC (0.7450), the SOTA model proved to be more robust and effective for breast tumor diagnosis (Table 2, Fig 4).

**Table 2:** The model performance on enhancement dataset.

Test	Model	AUC	Accuracy	F1	Precision	Recall
Internal	vgg16	0.8766	0.8231	0.6933	0.7222	0.6667
	Alex	0.8107	0.8231	0.6667	0.7667	0.5897
	dense	0.9329	0.8846	0.7945	0.8529	0.7436
	mobilnet	0.7236	0.7846	0.5758	0.7037	0.4872
	resnet	0.7921	0.8308	0.6452	0.8696	0.5128
	**sotaModel**	**0.9301**	**0.8692**	**0.7848**	**0.7750**	**0.7949**
External	vgg16	0.6459	0.6746	0.4875	0.6290	0.3980
	Alex	0.6821	0.6508	0.3231	0.6563	0.2143
	dense	0.7450	0.7262	0.6425	0.6526	0.6327
	mobilnet	0.6801	0.6508	0.3333	0.6471	0.2245
	resnet	0.6823	0.6310	0.4224	0.5397	0.3469
	**sotaModel**	**0.7776**	**0.7302**	**0.5952**	**0.7143**	**0.5102**

#### 3.2.3 Incorporation of Lesion Area Information.

To further enhance diagnostic performance, we incorporated image segmentation results by blending the radiologists’ annotated breast cancer ROI areas into the original image data for testing. The proposed state-of-the-art (SOTA) model demonstrated superior performance in both internal and external validation compared to other models. Internally, it achieved an AUC of 0.9980, accuracy of 97.69%, and precision of 1.0000, outpacing models like VGG16 (AUC: 0.8580, precision: 0.9500), AlexNet (AUC: 0.9065, precision: 0.8667), and DenseNet (AUC: 0.9566, precision: 0.9655). While DenseNet achieved the highest AUC, the SOTA model excelled in precision and recall, with a recall of 0.9231. Externally, the SOTA model achieved an AUC of 0.8438, accuracy of 74.60%, and precision of 0.6308, outperforming VGG16 (AUC: 0.7192, precision: 0.7941) and AlexNet (AUC: 0.7760, precision: 0.7241). Although DenseNet had a higher AUC (0.8234), the SOTA model displayed superior recall (0.8367), highlighting its robustness and effectiveness in breast tumor diagnosis across both internal and external datasets (Table 3, Fig 4).

**Table 3:** The model performance on dataset with lesion ROI.

Test	Model	AUC	Accuracy	F1	Precision	Recall
Internal	vgg16	0.8580	0.8385	0.6441	0.9500	0.4872
	Alex	0.9065	0.8692	0.7536	0.8667	0.6667
	dense	0.9566	0.9077	0.8235	0.9655	0.7179
	mobilnet	0.7464	0.7615	0.4746	0.7000	0.3590
	resnet	0.9121	0.8538	0.7246	0.8333	0.6410
	**sotaModel**	**0.9980**	**0.9769**	**0.9600**	**1.0000**	**0.9231**
External	vgg16	0.7192	0.6905	0.4091	0.7941	0.2755
	Alex	0.7760	0.7143	0.5385	0.7241	0.4286
	dense	0.8234	0.7659	0.7005	0.6970	0.7041
	mobilnet	0.6323	0.6071	0.3172	0.4894	0.2347
	resnet	0.7635	0.7143	0.5909	0.6667	0.5306
	**sotaModel**	**0.8438**	**0.7460**	**0.7193**	**0.6308**	**0.8367**

### 3.3 Clinical interpretability

To further validate the model’s effectiveness and clinical applicability, we employed Grad-CAM (Gradient-weighted Class Activation Mapping) for visualization analysis. Grad-CAM generates heatmaps to illustrate the regions focused on by the deep neural network during image classification, aiding in the interpretation of the model’s decision-making basis. In this study, we conducted a comparative visualization of the models under three different processing methods: original data, data augmentation, and the addition of lesion segmentation information.

**Fig 5:**
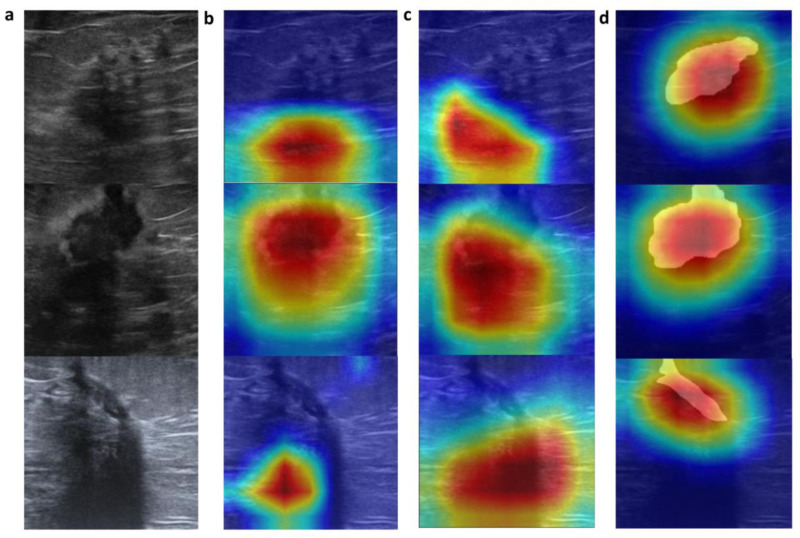
The Grad-CAM visualizations. a : Original data; b, c, d: Grad-CAM results under original data, data augmentation, and addition of lesion ROI processing methods, respectively.

The results showed that models without segmentation information performed poorly in localizing lesions, whereas models with added segmentation information accurately covered the lesion areas. In the attention results obtained from the three processing methods—original data, data augmentation, and addition of lesion ROI—the first two did not fully focus on the lesions. However, the model with added lesion ROI effectively covered the lesion areas (Fig 5).

These visualization results demonstrate that the model trained with integrated lesion ROI information can more reliably and accurately identify lesion areas. This further validates the model’s robustness and clinical applicability. The findings provide strong support for the model’s practical clinical use, indicating that integrating imaging segmentation information can significantly enhance the model’s diagnostic performance.

## 4 Discussion

This study proposes a state-of-the-art (SOTA) model based on a combination of MobileNet and ResNeXt for intelligent auxiliary diagnosis of breast tumors. By comparing with classic models such as VGG16, AlexNet, DenseNet, MobileNet, and ResNet, our SOTA model exhibited superior performance in multiple metrics, including AUC, accuracy, and F1 score, in both internal and external validations. The SOTA model achieved a best AUC of 0.92 and an accuracy of 83.84% in internal validation, while in external validation, it reached an AUC of 0.75 and an accuracy of 69.44%. These results indicate the high robustness and accuracy of the SOTA model in classification tasks. The lower accuracy on the external validation dataset highlights challenges in generalizing across clinical environments. To address this, we propose using transfer learning to adapt pre-trained models and federated learning for collaborative training while maintaining data privacy. These strategies could enhance the model’s performance for broader clinical use.

The superior performance of our model across various metrics can be attributed to its unique architectural design and adaptability. MobileNet’s depthwise separable convolutions significantly reduce the model’s parameters and computational complexity, maintaining high feature extraction capability while lowering resource consumption. The ResNeXt modules, with grouped convolutions, increase the network’s width, capturing fine details in the images more effectively. Furthermore, data augmentation and the integration of lesion segmentation information further enhanced the model’s performance, allowing it to handle complex medical images with greater accuracy and stability. These design choices confer a significant advantage to the hybrid model in multi-classification tasks for breast tumors.

This study has significant clinical implications. The proposed model enhances breast tumor diagnosis accuracy, reducing misdiagnoses and assisting radiologists in making precise decisions. By incorporating image segmentation, it provides more detailed diagnostic evidence, improving reliability and interpretability. Its efficiency and robustness make it suitable for resource-limited settings, supporting early breast cancer screening. Additionally, the model can reduce physicians’ workload and provide timely diagnoses, improving patient outcomes. However, challenges in clinical application may arise, such as data variability, image quality issues, and integration into existing workflows. Despite these, the model has the potential to advance breast cancer diagnostics and improve healthcare quality.

While this study achieved promising results in the diagnosis of breast cancer, several limitations need to be addressed. The current study focused primarily on the benign and malignant classification of breast tumors, and future work could broaden this scope to include additional types of breast diseases. Additionally, the test data used in this study were relatively limited, highlighting the need for multi-center validation to ensure the model’s applicability across diverse datasets and clinical environments. Moreover, although the model demonstrated effective diagnostic performance, it is not yet developed into a fully functional system for clinical practice. Future efforts should focus on further development and integration to enable clinical translation.

## 5 Conclusions

This study employs computer vision algorithms and various CNN models to model OSCC images. We designed a network based on Xception and multi-head attention mechanisms, demonstrating superior performance compared to other models. The proposed model can efficiently and rapidly complete diagnoses, providing timely and effective assistance to doctors in their diagnostic processes. This achievement holds the potential to become a rapid diagnostic tool, which, in the future, could be integrated with other deep learning-based diagnostic tools to offer tailored diagnostic and treatment plans based on patient data.
